# Environmental insults: critical triggers for amyotrophic lateral sclerosis

**DOI:** 10.1186/s40035-017-0087-3

**Published:** 2017-06-16

**Authors:** Bing Yu, Roger Pamphlett

**Affiliations:** 1 Sydney Medical School (Central), The University of Sydney, Camperdown, NSW 2006 Australia; 2 Department of Medical Genomics, Royal Prince Alfred Hospital and NSW Health Pathology, Camperdown, NSW 2050 Australia; 3 Discipline of Pathology, Brain and Mind Centre, The University of Sydney, 94 Mallett St, Camperdown, NSW 2050 Australia; 4 Department of Neuropathology, Royal Prince Alfred Hospital, Camperdown, NSW 2050 Australia

**Keywords:** Amyotrophic lateral sclerosis, Environmental risk factors, Mendelian randomisation, Mitochondrial dysfunction, Trigger, Initiation, Spread

## Abstract

**Background:**

Amyotrophic lateral sclerosis (ALS) is a fatal neurodegenerative disease characterised by a rapid loss of lower and upper motor neurons. As a complex disease, the ageing process and complicated gene-environment interactions are involved in the majority of cases.

**Main body:**

Significant advances have been made in unravelling the genetic susceptibility to ALS with massively parallel sequencing technologies, while environmental insults remain a suspected but largely unexplored source of risk. Several studies applying the strategy of Mendelian randomisation have strengthened the link between environmental insults and ALS, but none so far has proved conclusive. We propose a new ALS model which links the current knowledge of genetic factors, ageing and environmental insults. This model provides a mechanism as to how ALS is initiated, with environmental insults playing a critical role.

**Conclusion:**

The available evidence has suggested that inherited defect(s) could cause mitochondrial dysfunction, which would establish the primary susceptibility to ALS. Further study of the underlying mechanism may shed light on ALS pathogenesis. Environmental insults are a critical trigger for ALS, particularly in the aged individuals with other toxicant susceptible genes. The identification of ALS triggers could lead to preventive strategies for those individuals at risk.

## Background

Amyotrophic lateral sclerosis (ALS) is a rapidly progressive and universally fatal neurodegenerative disease of the human motor system. It is characterised by degeneration of upper (frontal motor cortex) and lower (spinal cord and brain stem) motor neurons, leading to progressive paralysis and death, usually due to respiratory failure. Its onset peaks around the mid-60s and most patients succumb to the disease within 2–5 years of becoming symptomatic [1, 2]. ALS is classically divided into familial and sporadic forms (Table 1), though these two forms are generally clinically and pathologically indistinguishable [1, 3]. The causes of sporadic ALS remain a research challenge [4]. Recent massively parallel sequencing technologies have facilitated disease-gene discovery, and rare variants in more than 50 genes have now been identified in association with sporadic ALS [5, 6].

**Table 1:** Genetic characteristics of familial and sporadic ALS

	Familial ALS	Sporadic ALS
Proportion	10%	90%
Disease category	Monogenic	Complex
Inheritance	Autosomal dominant (most common) Recessive X-linked	Gene-environment interactions Gene-gene interactions Autosomal recessive variants *De novo* variants
Common genes (% mutations)	*C9orf72* repeat expansions (24%) *SOD1* (20%) *TARDBP* (1–5%) *FUS* (1–5%) Other genes (rare or unknown)	*C9orf72* repeat expansions (5–10%) *SOD1* (1–3%) Other genes (rare or unknown)

*Abbreviations*: *C9orf72* chromosome 9 open reading frame 72 gene, *SOD1* superoxide dismutase 1 gene, *TARDBP* TAR DNA binding protein gene or TDP-43, *FUS* fused in sarcoma gene

**Fig. 1:**
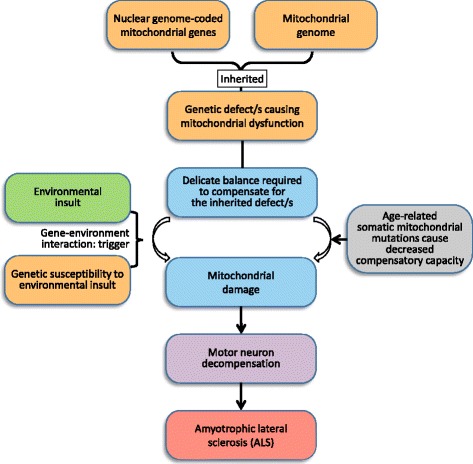
Proposed model of ALS pathogenesis. The primary defect is an inherited mitochondrial genetic abnormality, either from the nuclear genome-coded or mitochondrial genes. The mitochondria in the mutation carriers would be in a delicate balance with the struggle to compensate for such a defect. Compensatory capacity diminishes with ageing and the subsequent accumulation of somatic mitochondrial mutations. Environmental insults, particularly in the presence of genetic susceptibilities to toxicants, would further damage the mitochondria and trigger the decompensation process in motor neurons. Any attempt to compensate via excitatory transmission in the surviving neurons would increase their own metabolic load and adversely affect this delicate balance. These ineffective compensatory attempts then initiate a chain reaction of mitochondrial crisis and neuronal apoptosis, leading to ALS

The sporadic form of ALS in particular is considered to be complex disease (Table 1). A gene-time-environment model has been proposed, in which environmental risks and ageing interact with a pre-existing genetic load, followed by an unknown mechanism of self-perpetuating decline to death [1]. About 60% of the risk of sporadic ALS is genetically determined with the remaining 40% due to environmental factors [4, 7]. Advanced age and male gender are the only two established risk factors for ALS. Many environmental factors have been postulated for sporadic ALS (for comprehensive reviews see references [1, 8]), but none has been proven conclusively. Several critical issues remain unexplained, such as how sporadic ALS begins, how it spreads, and why it affects mostly motor neurons. In this article, we focus on several commonly postulated ALS-associated environmental insults that have support from Mendelian randomisation analyses. We also explore the consequences of gene-environment interactions, and how to further investigate the role of environmental factors in ALS. A model of ALS pathogenesis is proposed that links current knowledge of genetic factors and advancing age with the role of environmental factors (Fig. 1).

## Characteristics of the motor neuron system

### Motor neurons

Motor neurons, comprising the cell body, axon, dendrites and telodendria, coordinate voluntary actions and transmit signals to different muscles of the body. The axon is a long process which transmits nerve impulses over a long distance without diminution of the amplitude of the signal. For example, the motor neuron axon in the human sciatic nerve begins in the lumbar spinal cord, runs down the lower limb and reaches the foot. Dendrites form extensions that synapse with one or many other neurons and telodendria make contact with muscles at neuromuscular junctions.

### Astrocytes

Astrocytes represent 20–40% of the total number of cells in mammalian brains [9]. These glial cells form a network among neurons, dendrites and axons by means of their numerous processes radiating from their cell bodies, and creating functional units [10]. They are active partners of motor neurons and integrate or modify converging information through their contacts with neuronal synapses.

### Mitochondria

Mitochondria play multiple roles in calcium homeostasis, energy supply, metabolic synthesis and apoptosis [11]. There is a high density of mitochondria in motor neurons, including in the neuromuscular junction. The central nervous system consumes more energy than any other human organ, accounting for up to 20% of the body’s total use [12]. Approximately 90% of cerebral ATP production occurs in the mitochondria through oxidative phosphorylation [13, 14]. ATP utilisation occurs mainly in the axon that supports various cellular functions, including phospholipid metabolism, protein synthesis, neurotransmitter cycling, and transportation of ions across cellular membranes. Sodium, calcium and potassium ions are continuously and actively passed through the membranes of cells at the expense of ATP consumption, so that neurons can recharge to fire [13, 14].

The mitochondrial genome has about a 15 times higher mutation rate than that of the nuclear genome [11]. This could be related to its high concentration of reactive oxygen species, lack of protective histones, and limited DNA repair. Importantly for mitochondrial function, there are 1158 predicted mitochondrially-targeted proteins encoded by the nuclear genome [15]. Mitochondria play a central role in the complex balance of cellular processes contributing to ageing and neurodegeneration [11, 16] (Fig. 1). Interestingly, astrocytes act as recharging stations for neurons by supplying functional mitochondria [17].

## Environmental insults in ALS

It is becoming increasingly apparent that mutations in ALS-associated genes can lead to ALS. However, none of these mutations explains how the disease starts and spreads. The environmental contributions to the disease have been more challenging to uncover, in part because the lack of knowledge about disease mechanisms makes it difficult to determine which environmental insults to focus on.

### Infections

Viral and bacterial infections have been implicated as risk factors for ALS in several epidemiological and clinical studies [18–20]. Enteroviral nucleic acids have been identified in the spinal cords of sporadic ALS cases more frequently than controls [21–23]. Poliovirus, a member of the enteroviral family, affects motor neurons selectively [20] and individuals who have poliomyelitis in childhood may develop a progressive motor neuron disorder up to 40 years later, termed post-polio syndrome [24]. In vitro studies suggest that enteroviral infection of human motor neurons or glial cells can become persistent [25, 26]. This could have a significant impact on the motor neuron and result in altered astrocytic glutamate transport, decreased mitochondrial activity and impaired resistance to oxidative stress [27, 28] (Fig. 1). The lack of inflammatory change in the nervous tissue of most cases makes an acute viral attack on motor neurons unlikely. A persistent viral presence in neurons, however, may result in an atypical insult that could play a role in ALS.

The poliovirus receptor is a nuclear-encoded gene specific to the primate lineage, and serves as a cellular receptor for poliovirus in the first step of poliovirus replication. Its product is a transmembrane glycoprotein belonging to the immunoglobulin superfamily. The poliovirus receptor may be involved in the differentiation of motor neurons during embryonic development. Human poliovirus receptor variants can influence the consequence of poliovirus infection and possibly result in a persistent infection that later leads to ALS [29, 30].

Recently, the neural expression of latent human endogenous retrovirus group K (HERV-K) was detected in post mortem brain tissue from patients with sporadic ALS [31]. In vitro transfection of the HERV-K genome, or its env gene alone, into cultured human neurons can trigger neurite retraction and neuronal death. Transgenic mice expressing the HERV-K env gene showed abnormalities in intrinsic cortical hyperexcitability and impaired motor function, and 50% die by 10 months of age [31].

### Organophosphates

Organophosphates have been suspected as a risk factor in the pathogenesis of ALS due to their ability to damage motor neurons [32–34]. Chemicals containing organophosphates are present in fertilizers, herbicides, fungicides and insecticides and have wide agriculture and domestic usage [2, 35]. The toxicity of organophosphates is related to its acute inhibition of acetyl cholinesterase, the enzyme responsible for terminating the activity of the neurotransmitter acetylcholine. Chronic exposure to organophosphates can induce progressive brain damage by irreversibly inhibiting acetylcholinesterase, resulting in excessive simulation of cholinergic receptors and excitotoxicity [36]. Metabolites of various organophosphorus compounds [37] could also trigger neuronal damage and induce delayed neurotoxicity. Genetic susceptibility inhibiting organophosphate detoxification could be responsible for the reported association of pesticide with ALS, as will be discussed later.

An increase in ALS incidence has been reported in commercial pilots, navigators and flight attendants [33, 38]. Exposure to organophosphates has been proposed as the link in this group since engine air is supplied unfiltered to the aircraft cabin. This air can contain pyrolysed engine lubricating oils and hydraulic fluids through leaking oil seals or bearings, ruptured fluid lines, improper maintenance, or other malfunctions. Engine lubricating oils contain about 4% tricresyl phosphate [39], and hydraulic fluids contain other organophosphates, such as butyl phosphates [40].

### Heavy metals

Heavy metals including lead, mercury, cadmium and selenium have been implicated in the development of ALS [32, 35, 41, 42]. It is beyond the scope of this review to cover all individual metals, but aspects of mercury and lead are of interest. Mercury exists in a wide variety of physical states, elemental, organic, and inorganic. In an aquatic environment, elemental mercury undergoes biomethylation by bacteria and algae. The organic compounds that are obtained, such as methyl-mercury and ethyl-mercury, accumulate in fish, crustaceans, and throughout the food chain to humans. Mercury intoxication in the CNS disrupts cellular metabolism and degrades several cellular constituents, eventually leading to cell death and clinical disease. The biochemical mechanisms and the clinical pictures of mercury toxicity depend on individual genetic susceptibility, the chemical forms of the metal, and the length, and concentration of exposure.

An association between ALS and occupational exposure to lead has been proposed [43, 44]. Lead from human activities includes burning fossil fuels, mining and manufacturing. Workers exposed to welding or soldering materials appear to be at risk of developing ALS [45]. The toxic effects of lead on the nervous system include lead encephalopathy (primarily in children) and a motor neuropathy (primarily in adults). The half-life of lead is 1 month in blood, about 4 years in trabecular bones (such as the patella), and about 20 years in compact bone. Skeletal muscle can also be a storage site for lead [46]. Some sources of lead have been reduced in recent years, e.g., from gasoline, paints and ceramic products, caulking, and pipe solder [47], so it will be of interest to see if the incidence of ALS decreases in the future, as has been shown recently for Alzheimer disease in Western countries.

### Physical activity

Athleticism and intense physical activity have been considered important in ALS. A 6-fold increase in ALS has been reported in Italian professional footballers, with a dose-response relationship between the duration of playing and ALS risk [48]. Excessive physical activity, repeated head injuries, and exposure to pesticides and dietary supplements or illegal substances, could underlie the risk behind these footballers. Physical stress could enhance the production of reactive oxygen species, and it has further been suggested that exercise could increase the uptake of toxicants via the neuromuscular junction into human motor neurons [42]. Intense physical activity is characteristic of agricultural work and could be co-factor for ALS with exposure to organophosphates [34].

### Other environmental factors

Many other environmental factors have been investigated in relation to ALS, including organochlorines insecticides, pyrethroids, fumigants, smoking, electromagnetic fields, electric shocks, cyanotoxins and military service [8, 49]. Some persistent organic pollutants that originate from the past use of pesticides, solvents and industrial chemicals can also be risk factors for ALS. However, the available data are often conflicting. For example, organochlorines are associated with neurodegeneration in several Parkinson disease studies [50, 51], but their role in ALS is controversial. Exposure to aldrin, dieldrin, DDT (dichlorodiphenyltrichloroethane) and taxaphene tends to increase the odds ratios of ALS, but may be confounded by increasing age [49]. Smoking has been reported as a risk factor for ALS [52, 53], but the results are conflicting and lack of a clear dose-response relationship [1, 54]. The limitations of previous ALS epidemiological studies are discussed in the next section.

Military service represents a different category of risk factor since it aggregates a group of combined factors. Soldiers often received prophylactic treatment of cholinergic inhibitors to protect them against nerve gas and insect pests [55]. The deployment usually involves intensive physical activity, emotional stress and physical or psychological trauma, along with detrimental lifestyle factors such as cigarette smoking and alcohol consumption. Military personnel can also be exposed to environmental viruses, heavy metals, organophosphates, nasopharyngeal radium, exhaust from heaters or generators, high-intensity radar waves, contaminated food, explosions in the field [55, 56]. Finally, it has been suggested that exposure to diesel fuel, used extensively in the military, may underlie the increased risk of ALS in the services [56].

## Challenges in ALS epidemiology

### ALS could occur in number of different unique environments

Environmental risk factors for ALS have been studied for many years without any firm conclusions being drawn. Sporadic ALS is probably human-specific, since neurodegeneration affects neocortical regions and interconnections, the evolutionary consequence of *Homo sapiens*. Only higher primates have direct connections between upper and lower motor neurons, the two sets of neurons most affected by ALS. The extraordinary long motor axons with their complicated activities demand a high energy consumption. The complex natural, built and social environments that every human individual faces are unique to our species. For example, heavy metals can enter humans from breathing in particulate matter in the air, from drinking water with leached lead from pipes, or by eating accumulated mercury via the seafood chain. ALS animal models are different from humans as regards lifespan, with humans commonly living over 80 years but mice surviving only up to 3 years. An elderly human has therefore experienced vastly more cycles of mitochondrial DNA replication than an aged mouse, since the daily turnover of mitochondrial DNA is similar in mice and humans [16]. It is therefore not surprising that the use of animal models has proven less than fruitful for complex human disorders, particularly in relation to environmental risk factors.

### Limitations of investigating environmental risks in ALS

Environmental factors are widely considered to play a role in ALS pathogenesis. However, none of the known environmental risk factors has been conclusively determined [1]. Criticisms have been made on epidemiological study design and selection bias. Many previous investigations of the environmental effects on ALS have small sample and effect sizes, lack population controls and are retrospective in nature. Data collection largely relies on self-reporting through questionnaires or surveys with such studies being prone to recall bias. Misclassification of exposure may be responsible, for example, for the lack of concordance between survey data and measurements of blood pollutants [33]. Most of the published studies lack data on the frequency and intensity of toxicant exposure [57]. Furthermore, interpretation of the significance of environmental risk factors can be difficult in the absence of participants’ genomic information such as ALS susceptibilities.

It is difficult to assess how environmental insults initiate and influence the progression of ALS in observational studies. Even if an insult and an outcome are associated, the direction of causality can be hard to ascertain because ALS itself can obscure the intensity of an insult. For example, lead levels can be high in ALS, but that ALS itself can reversely affect the lead levels due to the release of bone and/or muscle-stored lead during osteoporosis and muscle wasting [46]. Strategies have been proposed to overcome these limitations. Prospective studies with population controls would be ideal, but hard to execute because they involve significant investment for detailed interviews, monitoring of environmental insults, and a long period of time to recruit sufficient numbers of patients with ALS which has modest incidence.

### Genetic proxies for environmental insults

**Fig. 2:**
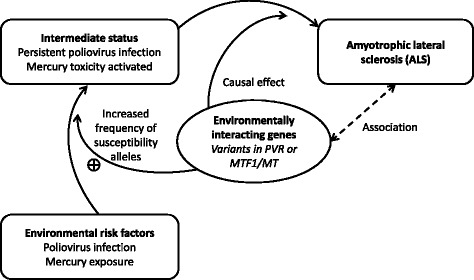
Genetic proxies for environmental insults, with examples given for poliovirus infection and mercury exposure. This illustrates the Mendelian randomisation approach to infer a causal role of environmental factors in ALS. ALS is associated with the environmental factors via an intermediate status, and the environmental interacting genes (*dashed line*). But the effect of the interacting genes is directional and facilitates the establishment of the intermediate status. Such an effect can only occur in the presence of environmental insults. The susceptibility alleles of the interacting genes will not be reversely affected by the ALS outcome since all genotypes are determined at conception

The application of Mendelian randomisation can shed light on causal relationships since this uses a genetic proxy (e.g., single nucleotide variants, SNVs) as a variable to assess environmental exposure (Fig. 2) [58, 59]. The principle of Mendelian randomisation is based on Mendel’s second law of independent assortment, i.e., the random assortment of genes from parents to offspring that occurs during gamete formation and conception. This implies that SNVs will not be associated with any confounding factors that may distort conventional observational studies at a population level, and such variants are unlikely to be affected by reverse causality since the genotypes are determined at conception. For a SNV to be a valid instrumental variable, it must be reliably associated with the exposure, and only be associated with the outcome through the exposure of interest (Fig. 2). Such variants should be independent of other factors affecting the outcomes [60–62]. This strategy can be used for ALS research with two related purposes: (1) to provide evidence for the existence of causal associations, and (2) to enable accurate estimation of the magnitude of the effect of lifelong exposure to an environmental insult. If environmental factors are truly a causal risk factor in ALS development, their susceptible genetic proxies would be expected to increase risk of ALS. Three examples of this strategy are given below.

1. Poliovirus is a lytic virus, but it can also establish a persistent infection like other enteroviruses [25, 30]. SNVs in the human poliovirus receptor gene can influence the consequences of the infection. For example, 56% of wild-type human neuroblastoma cells survived 28 h after poliovirus infection, but survival increased to 79% in cells with a particular SNV (rs1058402) [29]. When 110 patients with sporadic ALS and 30 with progressive muscular atrophy (PMA, the lower motor variant of ALS) were compared with 280 controls, the frequency of SNV rs1058402 was 20% in PMA cases and 12% in ALS cases, significantly higher than in controls at 7% [63]. These results support a pathogenic role of enteroviruses in ALS since affected neurons could survive the early cytolytic effect of poliovirus and establish the persistent infection. The expression of poliovirus receptor is weak in spinal motor neurons and strong in muscle motor endplates, suggesting that neuromuscular junctions serve as routes for viral entry into lower motor neurons [64]. A slowly accumulative cytopathic effect on spinal motor neurons along with ageing could then trigger ALS (Figs. 1 and 2). This risk factor could be target for treatment since persistent enterovirus infection can be treated with antiviral agents [65].
2. Organophosphates are activated to their reactive oxons by the cytochrome P450 system, and these oxons are hydrolysed by paraoxonase, which is encoded by *PON1*. PON1 has different levels of hydrolytic enzyme activity and such variation is genetically determined [66]. Rare *PON 1* variants or haplotypes that lead to a decrease in paraoxonase activity are associated with ALS [67, 68]. Genetic variants that reduce the ability of the body to detoxify organophosphates could therefore favour ALS development. However, the association of *PON 1* with ALS has failed to be reproduced in a large meta-analysis [69].
3. Metallothioneins (MT) are metal-binding proteins involved in the detoxification of heavy metals such as mercury. A lack of MT increases heavy metal toxicity (Fig. 2), while overexpression is protective [70]. Metal transcription factor-1 (*MTF1*) acts as a sensor and regulator for MT expression and upregulation in response to heavy metals [71] and any change in *MTF1* could disrupt the upregulation of MTs and leave motor neurons vulnerable to heavy metal damage (Fig. 2). When the relevant genetic variants in the *MT* gene family and *MTF1* were studied in 186 sporadic ALS cases and 186 controls, significant differences were found in the distribution of some SNVs in MT detoxification and *MTF1* genes between the cases and controls [72]. Less efficient metal detoxification could therefore be a risk factor for ALS.

## The role of mitochondria in ALS

### How is ALS initiated?

Much evidence implicates mitochondrial dysfunction in ALS. Mitochondrial shape and positioning in cells is crucial for bioenergetics [11]. Morphological changes observed in ALS mitochondria in the anterior horn of the spinal cord include smaller size, disrupted crests and edema, crystolysis and vacuolisation, indicating metabolic disturbances [73]. These changes were unlikely to be the artefacts due to ageing or post mortem process because they were significantly different from 15 age-matched control samples. Interestingly, similar changes can be found in liver and muscle cells [74, 75], supporting the concept that mitochondrial defects are inherited from either mitochondrial or nuclear genomes [74, 75] (Fig. 1).

Mitochondrial mutations are maternally inherited or result from somatic changes. These mutations can progressively increase with age through neural clonal expansion [76] (Fig. 1). High metabolic rate and ATP consumption make the human motor system particularly vulnerable to energy deficiency. Included in the 126 ALS genes in the Amyotrophic Lateral Sclerosis Online genetics Database (v6) are one mitochondrial gene (MT-ND2) and 10 nuclear genome-coded mitochondrial genes (*ATXN2, CHCHD10, GARS, MAOB, OGG1, OMA1, PARK7, SOD1 SOD2* and *SPG7*), found when ALS genes are cross-over with MitoCarta [5, 15]. Other mitochondrial variants, particularly nuclear genome coded ones, could be misinterpreted as variants of unknown significance due to their population frequencies or less clear-cut functionality. A study of 44 ALS case-unaffected parents trios found that ALS can be transmitted in an autosomal recessive way by homozygous or compound heterozygous changes [6] (Table 1). The frequencies of ALS-related recessive alleles could therefore be higher than expected and be adversely filtered out due to the cut-off values used in the whole exome or whole genome studies.

The finding that mitochondria can be transferred from astrocytes to neurons supports the critical role of mitochondria in neurons, and the possible involvement of astrocytes in ALS pathogenesis [17, 77]. This is of interest since mercury, long suspected in the pathogenesis of ALS, first enters the CNS via uptake by perivascular astrocytes, and is found predominantly within mitochondria [78]; any transfer of mercury-laden mitochondria from astrocytes into motor neurons could result in neurotoxic damage to these neurons. Furthermore, reduced mitochondrial content with age and somatic changes in post-mitotic neurons could lead to a decline of mitochondrial function [79].

ALS-susceptible mitochondrial variants are unlikely to remain ‘switched off’ until mid- to late-adulthood, although they may not be sufficient to cause any overt mitochondrial disease in early life. The motor neuron system in these individuals could be in a delicate balance with a constant struggle to compensate for such a defect or defects (Fig. 1). Compensatory capacity diminishes with ageing and the compromised mitochondrial function may finally collapse. Environmental insults could further affect the mitochondria, particularly in the presence of susceptibility alleles of the interacting genes, and trigger the decompensation process in ALS susceptible individuals (Figs. 1 and 2). It is also possible that astrocytes with mitochondrial defects may be the target cells for the harmful action of some environmental insults (such as mercury, see above), while neuronal death may be a secondary event following the initial insult to astrocytes closely related with motor neurons [10, 77].

Persistent viral infection, organophosphates, heavy metals and intense physical exercise could put metabolic loads on defective mitochondria and exhaust any compensatory capacity (Fig. 1). Other mechanisms including excitotoxins, oxidative stress, or altered calcium homeostasis could participate in cell damage [77]. Disease triggers could be disguised as the root cause of ALS, and generate conflicting results in environmental studies, since these initial insults may be necessary but not sufficient for the pathogenesis of ALS. Environmental insults, even with similar intensity and exposure time, are unlikely to have similar impacts on non-susceptible individuals.

### ALS spread

Any loss of motor neurons would put extra stress on surviving motor neurons that innervate the same muscle and increase the metabolic needs to compensate for the loss. Astrocytes at this stage may fail to perform the normal maintenance to axons or neuronal cell bodies since they would divert their resources in attempts to rescue decompensating neurons. As a consequence, more neurons would enter the decompensating process. A decrease in motor unit number and an increase in cortical excitability is found before symptom onset in *SOD1* mutation carriers [80, 81]. Such excitatory compensation may not be helpful, but instead initiate a chain reaction of mitochondrial crisis and neuronal apoptosis. Excitotoxicity can increase calcium flow into the neuron, initiate oxidative stress, and result in neuronal death. A mitochondrial crisis could also influence proteasomal or autophagic protein degradation and amplify the cellular stress. Environmental risk factors such as muscle-stored heavy metals released during muscle wasting [46] could further accelerate the deterioration. Of note, a recent study has shown how human spinal interneurons, which normally inhibit motor neurons, take up heavy metals during ageing; any mercury within the mitochondria of these interneurons could lead to interneuron malfunction with subsequent excitotoxicity to motor neurons [82].

This proposed model could explain the well-known clinical and pathological pattern of ALS starting in one CNS region and ‘spreading’ to other adjacent region [83]. This spread may be due to a cascade of decompensating neurons. This model therefore avoids the presumption that any environmental agent travels from one neuron to another through their synapses, extracellular vesicles, or membrane contacts. The proposed model provides a unique mechanism involving a decompensation process for spreading and “gain of toxic strength” for the subsequent accelerated progression of ALS.

### Association of the proposed model with known ALS features

The development of ALS has been considered as involving a six-step process [84]. Further identification of these steps could lead to novel preventive or therapeutic avenues. Our proposed model is consistent with the gene-time-environment hypothesis [1] and entails multiple steps (Fig. 1). It offers a potential single root of ALS pathogenesis, with environmental insults being a trigger for ALS initiation. The available evidence has suggested that primary inherited defect(s) could cause mitochondrial dysfunction that establishes the susceptibility of motor neurons to ALS (Fig. 1). Environmental insults then upset the delicate balance of mitochondrial function, followed by propagation and acceleration due to an ineffective compensating process. Metal homeostasis is intimately coupled to the oxidative stress response in many cell types [71]. The depletion of microtubules and neurofilaments in ALS motor neurons could result from the genetic predisposition. Consequently, it would impair normal transport and affect mitochondrial function due to lack of sufficient nutrients [85]. Environmental insults can also trigger adverse responses such as neuroinflammation that include activation of astrocytes and microglia, as well as direct motor neuron toxicity.

Persistent viral infection could be one environmental trigger of the decompensation process. It is unlikely that the relevant virus could be isolated, or any serological reaction be sufficiently generated, though microbiome studies of CNS tissue and muscle would be of interest. The model explains the paradox of the concept of virus spreading from one neuron to another with no evidence of any viral presence. Rather, the cellular stress of one neuron could be spread to activate the endogenous retrovirus in the neighbouring neurons via the expression of the env protein [31].

As suggested by Mendelian randomisation analyses, some ALS patients would have less efficient abilities to detoxify heavy metals, which could be enough to tip motor neurons beyond the point of sustained viability, resulting in the initiation of motor neuron loss and the decompensation process. Interestingly, loss of mobility and innervated nerve stimulation to muscle can accelerate the decompensation process, since more heavy metal such as lead can be released due to osteoporosis and loss of muscle bulk [46].

The proposed model emphasises gene-environment interactions, which involves multiple steps. Some crucial environmental insults might have arisen in early development, which makes them difficult to identify. For example, subclinical enterovirus or poliovirus infection, or heavy metal exposure, could occur early in life and only play a role in the initiation or acceleration stage of the disease in later life. Differently-susceptible individuals could inherit different genetic defects with different impacts on mitochondrial function and require different intensities of environmental triggers. Major inherited defects in mitochondria-related genes may only need occult or mild triggers, while other inherited variants may require a combination of environmental insults, e.g., military deployment, to evoke the onset of ALS.

## Conclusion

The available evidence has suggested that inherited defect(s) could cause mitochondrial dysfunction, which would establish the primary susceptibility to ALS. Further study of the underlying mechanism may shed light on ALS pathogenesis. Environmental insults are a critical trigger for ALS, particularly in the aged individuals with other toxicant susceptible genes. The identification of ALS triggers could lead to preventive strategies for those individuals at risk.

## Abbreviations

ALS: Amyotrophic lateral sclerosis
HERV-K: Human endogenous retrovirus Group K
SNV: Single nucleotide variants
